# Case Report: Dramatic response to amivantamab plus chemotherapy in EGFR G719A–mutant lung adenocarcinoma with liver and peritoneal metastases

**DOI:** 10.3389/fonc.2026.1820018

**Published:** 2026-05-26

**Authors:** Jiwon Kim, Han Gyeol Kim, Seung Hyeun Lee

**Affiliations:** 1 Division of Pulmonary, Allergy, and Critical Care Medicine, Department of Internal Medicine, Kyung Hee University College of Medicine, Kyung Hee University Hospital, Seoul, Republic of Korea; 2 Department of Pathology, Kyung Hee University College of Medicine, Kyung Hee University Hospital, Seoul, Republic of Korea; 3 Department of Precision Medicine, Graduate School, Kyung Hee University, Seoul, Republic of Korea

**Keywords:** amivantamab, chemotherapy, EGFR G719A, lung cancer, resistance

## Abstract

**Background:**

Hepatic metastasis and uncommon mutations are recognized risk factors in patients with epidermal growth factor receptor (EGFR)-mutant non–small cell lung cancer. The phase 3 MARIPOSA-2 trial demonstrated superior outcomes with amivantamab plus chemotherapy compared to chemotherapy alone in patients whose disease progressed during EGFR-tyrosine kinase inhibitor therapy. However, its efficacy and tolerability in patients with hepatic metastases or uncommon EGFR mutations remain unclear.

**Case description:**

A 52-year-old male smoker was diagnosed with stage IVB lung adenocarcinoma harboring an uncommon EGFR G719A mutation. He received afatinib as first-line therapy, followed by pemetrexed/carboplatin and later a quadruple regimen of atezolizumab, bevacizumab, paclitaxel, and carboplatin. Despite these treatments, disease progression occurred, presenting as multiple hepatic metastases, massive ascites, and new brain lesions, resulting in declining performance status. A combination of amivantamab, pemetrexed, and carboplatin was initiated as fourth-line salvage therapy.

**Results:**

Follow-up imaging showed marked regression of the lung mass, hepatic and brain metastases, and resolution of ascites, suggesting a near-complete response. His performance status gradually improved, and the response has remained durable for over nine months with manageable grade 1–2 skin rash and stomatitis.

**Conclusion:**

This case is the first to demonstrate a durable, near-complete response to amivantamab combined with chemotherapy in a patient harboring an uncommon EGFR G719A mutation and extensive visceral metastases. Although further studies are warranted, this report highlights amivantamab-based combination therapy as a promising salvage option for high-risk patients.

## Introduction

Although epidermal growth factor receptor (*EGFR*) tyrosine kinase inhibitors (TKIs) have shown survival benefits in patients with EGFR-mutant non–small cell lung cancer (NSCLC), resistance to TKIs is inevitable in almost all cases (1). In the event of first-line TKI failure, cytotoxic chemotherapy has long been used as a subsequent treatment; however, its efficacy remains suboptimal, with a progression-free survival (PFS) of approximately 4–6 months (2, 3). Recently, the phase 3 MARIPOSA-2 trial demonstrated superior clinical benefits of amivantamab combined with chemotherapy compared with chemotherapy alone in patients who had progressed after prior osimertinib treatment and harbored common EGFR mutations, including exon 19 deletion and exon 21 L858R (4). Notably, in patients with a history of brain metastases, the combination of amivantamab and chemotherapy yielded superior intracranial PFS compared with chemotherapy alone, with a hazard ratio (HR) of 0.55 (95% CI, 0.38–0.79) (4). Therefore, amivantamab combined with chemotherapy is now recommended as a subsequent treatment option for patients with EGFR-mutant NSCLC who experience progression during EGFR- TKI therapy (5).

Uncommon EGFR mutations constitute approximately 10% of all EGFR mutations and remain a heterogeneous group with limited treatment options, often exhibiting reduced sensitivity to first- and third-generation TKIs compared with common mutations (6). Although afatinib and osimertinib are recommended for this patient population, their efficacy is suboptimal, and data on subsequent treatment strategies are very limited (7, 8). Hepatic metastasis is another well-known risk factor in patients with lung cancer across various therapeutic settings, including targeted therapy and immunotherapy (9). A recent subgroup analysis of phase 3 MARIPOSA trial demonstrated superior efficacy of first-line combination using amivantamab plus lazertinib to osimertinib alone in EGFR-mutant NSCLC with hepatic metastasis (3). However, the efficacy of amivantamab-based combination regimens in patients with hepatic metastases who have failed prior EGFR-TKI therapy remains largely unknown.

We recently observed a dramatic and durable response to amivantamab plus chemotherapy in a heavily pretreated patient with EGFR G719A–mutant NSCLC and disseminated disease, including hepatic metastasis and peritoneal seeding. This case highlights the clinical activity of dual EGFR–MET inhibition in managing the biological aggressiveness associated with uncommon mutations and extensive visceral involvement. These findings provide supportive evidence that amivantamab-based regimens may represent a feasible therapeutic strategy for this high-risk patient population.

## Case presentation

**Figure 1:**
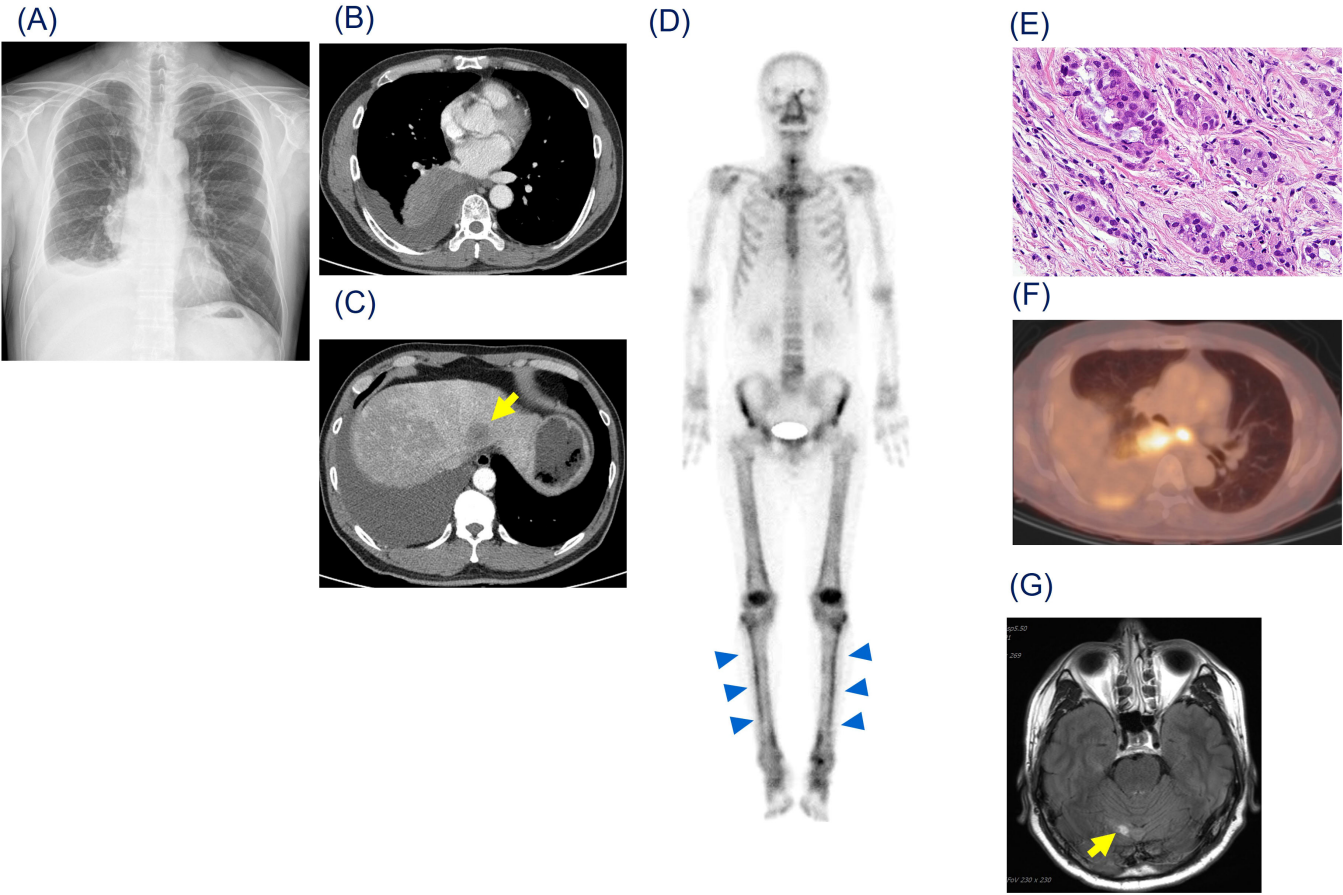
Imaging findings at diagnosis. The initial chest radiograph shows pleural effusion in the right hemithorax **(A)**. A chest computed tomography (CT) scan reveals a large mass in the right lower lobe with associated pleural effusion **(B)**, while an abdominal CT scan demonstrates a metastatic lesion in the left hepatic lobe (arrow, **C**). Although there was no evidence of bone metastasis on the bone scan, symmetric periosteal uptake along both fibulae, consistent with hypertrophic osteoarthropathy, was observed (arrow heads, **D**). A biopsy of the lung mass confirmed primary lung adenocarcinoma (hematoxylin and eosin staining, ×400) **(E)**. Positron emission tomography identified metastases in the right pleura and mediastinal lymph nodes **(F)**, and a brain magnetic resonance imaging scan revealed a metastatic lesion in the upper cerebellum (arrow, **G**).

A 52-year-old man presented to the Division of Rheumatology at our institution in September 2021 with a one-month history of bilateral leg pain and knee arthralgia. He had no significant past medical history and was not taking any regular medications but had a 15 pack-year smoking history. On physical examination, breath sounds were clear, but digital clubbing was noted. Chest radiography revealed pleural effusion in the right hemithorax (**Figure 1A**). A chest computed tomography (CT) scan showed an 80-mm mass obstructing the superior segmental bronchus of the right lower lobe (RLL) and confirmed pleural effusion in the right hemithorax (**Figure 1B**). An abdominal CT scan revealed a metastatic lesion in the left lobe of the liver (**Figure 1C**, arrow). A bone scan performed to evaluate his leg pain demonstrated symmetric linear uptake along both fibulae, consistent with hypertrophic osteoarthropathy (**Figure 1D**, arrowheads). He was referred to pulmonary division for further evaluation of suspected lung cancer. We drained the pleural effusion using percutaneous catheter insertion, and analysis of the drained fluid revealed a lymphocyte-dominant exudate without elevation of adenosine deaminase. Cytologic examination of the effusion confirmed metastatic carcinoma. A biopsy obtained from the RLL mass also demonstrated lung adenocarcinoma (**Figure 1E**). Positron emission tomography (PET) confirmed metastases involving the subcarinal lymph node and right pleura (**Figure 1F**), while brain magnetic resonance imaging (MRI) revealed three subcentimeter metastatic lesions in the right frontal lobe, right occipital lobe, and superior cerebellum (**Figure 1G**, arrow). Accordingly, the final clinical stage was determined to be stage IVB (cT4N2M1c) according to the criteria of the American Joint Committee on Cancer 8th edition. The tumor proportion score for programmed death-ligand 1 expression using the SP263 clone was 0%. Molecular analysis employing a peptide nucleic acid clamping–based real-time polymerase chain reaction (PCR) assay (PANAMutyper™, PANAGENE, Seoul, South Korea) detected an *EGFR* G719A mutation in exon 18. Next-generation sequencing (NGS) of the primary tumor tissue was performed using a targeted gene panel (Oncomine Comprehensive Assay Plus™, Thermo Fisher Scientific, Waltham, MA, USA), which confirmed the same mutation without any concurrent genetic alterations. Based on these findings, we initiated afatinib 40 mg daily as first-line therapy and prescribed celecoxib and acetaminophen to control leg and knee pain.

**Figure 2:**
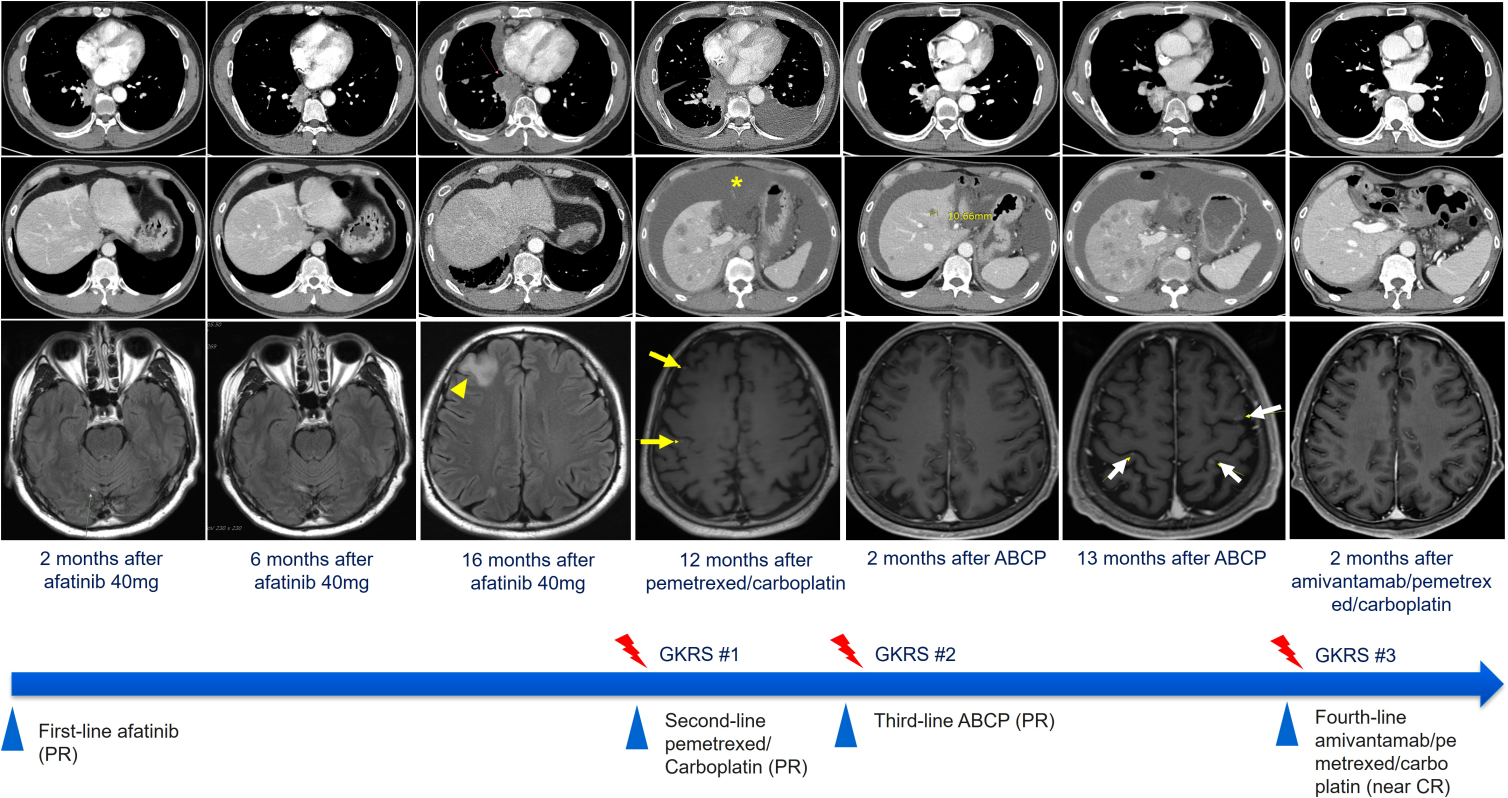
Clinical course with subsequent treatments. The first response evaluation with chest CT and brain MRI showed a marked tumor reduction after first-line afatinib therapy. The response was maintained for 16 months during afatinib treatment. However, pleural metastases progressed, and a new brain metastasis emerged in the right frontal lobe (yellow arrow). Follow-up EGFR testing did not detect the T790M mutation; therefore, the treatment regimen was switched to pemetrexed plus carboplatin, and gamma-knife radiosurgery (GKRS) was performed for the brain lesion. The second-line treatment was effective for 12 months, as evidenced by follow-up chest CT and brain MRI findings. The patient later presented with dyspnea and abdominal pain, and chest and abdominal CT scans revealed markedly aggravated pleural and hepatic metastases with massive ascites, cytologically confirmed as carcinoma peritonei. Brain MRI also showed aggravated brain metastases (arrow). As third-line therapy, a combination of ABCP was initiated along with a second GKRS. This regimen was effective, as demonstrated on CT and brain MRI, and the duration of response lasted 13 months. However, peritoneal seeding and hepatic and brain metastases subsequently recurred, and his performance status deteriorated accordingly. After multidisciplinary counseling with his family, salvage treatment with amivantamab, pemetrexed, and carboplatin was initiated, together with a third GKRS. Remarkably, all metastatic lesions—including those in the liver, peritoneum, and brain—completely resolved in follow-up imaging performed two months later, indicating a complete remission. The response has been sustained for more than nine months, and the patient continues amivantamab and pemetrexed therapy without significant adverse events. ABCP, atezolizumab, bevacizumab, paclitaxel, and carboplatin; PR, partial response; CR, complete response.

During first-line treatment, the patient experienced diarrhea, acneiform dermatitis, and stomatitis (all grade 1), which did not require dose reduction; therefore, full-dose therapy was maintained. **Figure 2** summarizes the chronological imaging changes observed during the clinical course according to different treatment regimens. The first response evaluation CT scan, performed two months after treatment initiation, demonstrated decreased size of RLL mass, pleural effusion, hepatic lesion, and brain metastases. His leg and knee pain resolved completely, allowing discontinuation of analgesics. The response was maintained without aggravation of adverse events (AEs). After 16 months of afatinib treatment, follow-up CT revealed regrowth of the RLL mass, and brain MRI showed a new metastatic lesion with peritumoral edema in the right frontal lobe (**Figure 2**, yellow arrowheads). Rebiopsy of the lung mass and liquid biopsy using plasma both detected only the EGFR G719A mutation without the T790M resistance mutation. Accordingly, we switched the regimen to pemetrexed plus carboplatin in accordance with clinical guidelines, while performing gamma knife radiosurgery (GKRS) for the brain lesion. The first response-evaluation CT scan after two treatment cycles showed a partial response, and pemetrexed was continued as maintenance therapy for the next 10 months. One day during the treatment, the patient presented with dyspnea and abdominal distention. Chest and abdominal CT revealed increased bilateral pleural effusion, ascites (**Figure 2**, asterisk), and multiple hepatic metastases. Cytologic analysis of ascitic and pleural fluids confirmed pleural and peritoneal seeding, and brain MRI also identified new metastatic lesions (yellow arrow), consistent with systemic disease progression. EGFR testing using PCR did not detect the T790M mutation, and NGS of the ascitic fluid revealed the original G719A mutation without any additional genomic alterations. Therefore, we performed another session of GKRS and changed the regimen to a combination of atezolizumab, bevacizumab, paclitaxel, and carboplatin (ABCP) (10). The response evaluation CT scan obtained after four treatment cycles showed a marked decrease in most lesions, including RLL mass, pleural effusion, ascites, and hepatic metastases. We maintained the treatment using atezolizumab and bevacizumab as maintenance therapy. Thirteen months after initiating this regimen, the patient presented with abdominal distention, anorexia, and generalized weakness. Follow-up CT imaging revealed an increase in the RLL mass but markedly aggravated multiple hepatic metastases and massive ascites. Brain MRI also demonstrated newly developed multiple brain metastases (**Figure 2**, white arrows). His Eastern Cooperative Oncology Group performance status (PS) had worsened to 3, suggesting that further chemotherapy could deteriorate his condition rather than provide clinical benefit. During two days of draining 6 L of ascitic fluid for symptomatic relief, we discussed potential therapeutic options and their risks and benefits with his wife. Finally, we decided to initiate a combination regimen of amivantamab plus pemetrexed and carboplatin as fourth-line treatment, with the dosage and administration schedule following the protocol of the phase 3 MARIPOSA-2 trial. Concurrently, a third session of GKRS was performed for the brain lesions. During the first month of treatment, his abdominal distention, general symptoms, and PS gradually improved. Remarkably, CT and brain MRI performed two months after treatment initiation revealed a profound systemic response, characterized by marked shrinkage of the primary lung mass and multiple hepatic metastases, alongside the complete resolution of both ascites and brain metastases. These findings were consistent with a near-complete response. Although grade 2 neutropenia and thrombocytopenia developed after the first cycle of the treatment, these adverse events were well managed with granulocyte colony-stimulating factor administration and dose reduction of the chemotherapeutic agents. Regarding AEs related to amivantamab, the patient did not experience infusion-related reactions and presented only with grade 1 stomatitis, grade 1 paronychia, and grade 2 acneiform rash on the face, which were well controlled using oral doxycycline and topical corticosteroids. Encouragingly, the response has remained durable for the past seven months without compromising his PS. He is currently maintaining amivantamab and pemetrexed therapy without interruption or further dose reduction, with a total treatment duration of nine months to date.

## Discussion

Although amivantamab plus chemotherapy has recently been approved as a later-line treatment for EGFR-mutant NSCLC, data regarding its efficacy and tolerability in patients with hepatic metastases or those harboring uncommon EGFR mutations remain limited. To the best of our knowledge, this is the first report demonstrating a rapid and systemic response to this combinational approach in a patient with an uncommon EGFR mutation and disseminated disease involving the liver and brain. This case highlights that such a regimen could represent a feasible and valuable therapeutic option for patients with EGFR-mutant NSCLC, even those with high-risk clinical features.

EGFR-TKIs have revolutionized the treatment landscape of EGFR-mutant lung cancer. First- and second-generation EGFR-TKIs have prolonged PFS to approximately 9–15 months (11, 12), while third-generation agents, including osimertinib and lazertinib, have further extended PFS to 18.9 months and overall survival (OS) up to 36 months (13, 14). Moreover, first-line combination therapy using osimertinib plus chemotherapy has recently demonstrated a notable survival benefit, achieving an OS of approximately 48 months (15). However, the emergence of resistance to TKIs is inevitable in nearly all patients, posing a major challenge in clinical management. Evidence indicates that resistance develops through either on-target or off-target mechanisms. The former includes the development of secondary EGFR mutations, such as T790M or C797X, whereas the latter encompasses mesenchymal–epithelial transition (MET) amplification, bypass pathway activation, and small-cell transformation (1). Unlike the resistance mechanisms observed with first- and second-generation EGFR-TKIs—where the T790M mutation accounts for approximately 50% of cases—resistance to third-generation EGFR-TKIs is more heterogeneous and complex. Among these mechanisms, MET amplification accounts for approximately 15–30% and is thus considered a major target for overcoming resistance in this clinical scenario (16).

Amivantamab is a fully human IgG1 bispecific antibody that targets both EGFR and MET receptors. Unlike traditional small-molecule TKIs, amivantamab exerts its antitumor activity through a multipronged mechanism (17). Its key actions include receptor downregulation through induced internalization and lysosomal degradation of EGFR and MET. By binding to the extracellular domains of these receptors, amivantamab can overcome resistance conferred by traditional acquired mutations (e.g., T790M or C797S). Moreover, preclinical models have demonstrated that amivantamab also induces immune-mediated antitumor effects. Specifically, its low-fucose Fc region enhances affinity for FcγRIIIa, thereby promoting antibody-dependent cellular cytotoxicity (ADCC) via natural killer (NK) cells and trogocytosis mediated by monocytes and macrophages (18, 19). Amivantamab has been approved for the treatment of patients with advanced NSCLC harboring EGFR exon 20 insertions, either as monotherapy or in combination with chemotherapy, based on results from the CHRYSALIS and PAPILLON trials (2, 20). More recently, the combination of amivantamab plus chemotherapy was approved for patients with EGFR-mutant NSCLC who had progressed during prior osimertinib treatment, as demonstrated in the MARIPOSA-2 trial (4). However, data remain lacking regarding whether this combinational regimen is both tolerable and effective in patients with disseminated disease and high tumor burden, or in those harboring uncommon EGFR mutations.

In the present case, amivantamab combined with chemotherapy elicited a dramatic clinical response in a heavily pretreated patient with EGFR G719A–mutant NSCLC who had extensive hepatic metastases, peritoneal seeding, and brain metastases. In the MARIPOSA-2 study, amivantamab plus chemotherapy demonstrated favorable intracranial efficacy: it reduced the risk of intracranial progression by 45% (HR 0.55; p = 0.001), extending the median time to central nervous system progression to 12.5 months versus 8.3 months for chemotherapy alone. Moreover, the intracranial objective response rate more than doubled—55% in the amivantamab arm compared with 27% in the chemotherapy control group (4). These findings support the expectation of a potential intracranial response in our patient.

More importantly, this case is notable in that disseminated hepatic metastases and peritoneal seeding markedly improved following the initiation of amivantamab-based combination therapy. Hepatic metastasis is a well-established poor prognostic factor in patients with lung cancer across various therapeutic settings, including targeted therapy and immunotherapy (9, 21). Although the underlying mechanisms linking hepatic metastasis and treatment resistance are not yet fully elucidated, activation of bypass signaling pathways and enhanced immune evasion are believed to play major roles (22). Although no direct evidence currently supports the efficacy of amivantamab plus chemotherapy in patients with hepatic metastasis, prior data suggest that amivantamab-containing combinations may represent a feasible therapeutic option in such clinical scenarios. In the phase 3 MARIPOSA trial, first-line amivantamab plus lazertinib demonstrated superiority over osimertinib in patients with hepatic metastases, significantly improving median PFS (18.2 vs. 11.0 months; HR 0.58; 95% CI, 0.44–0.77) and yielding a higher objective response rate (ORR; 83% vs. 63%) among patients with baseline hepatic metastases (23).

The underlying biological mechanisms through which amivantamab exerts its potent antitumor activity in the context of hepatic metastases remain incompletely understood. However, this activity is likely attributable to its bispecific structure and immune-modulating properties. Hepatic progression in EGFR-mutant NSCLC is often driven by MET-mediated bypass signaling (22). Amivantamab’s dual extracellular blockade of both EGFR and MET effectively suppresses these compensatory pathways. Moreover, its capacity to induce ADCC and trogocytosis may exploit the dense population of resident immune cells in the liver—such as Kupffer and NK cells—to enhance tumor cell clearance (18, 19). Further research is warranted to elucidate the precise molecular mechanisms of amivantamab and to explore potential synergistic effects when combined with TKIs or chemotherapy in the treatment of hepatic metastases.

Another noteworthy finding in our case is that the combination of amivantamab plus chemotherapy was effective in a patient harboring an uncommon EGFR mutation. Uncommon EGFR mutations account for approximately 10–20% of all EGFR-mutant NSCLC cases and exhibit high heterogeneity. These mutations are generally considered adverse prognostic factors because they are associated with poorer responses to EGFR-TKIs compared with common mutations (8, 24). According to current guidelines, afatinib and osimertinib are recommended as first-line treatments for several uncommon mutations, including S768I, L861Q, and G719X (25). In a *post hoc* analysis of data from three clinical trials involving patients with uncommon EGFR mutations, afatinib demonstrated meaningful efficacy, with an ORR of 71% and a median PFS of 10.7 months (8). Similarly, in a prospective study including 40 patients with these mutations, osimertinib showed favorable outcomes, achieving an ORR of 55% and a median PFS of 9.4 months (24). However, in that study, osimertinib demonstrated a lower ORR in patients with G719X mutations than in those harboring L861Q mutations (30% vs. 86%) (24). Based on these findings, we selected afatinib as first-line therapy and achieved a relatively durable response lasting 16 months, which exceeds the duration reported in previous studies (8, 25).

Although evidence remains limited, recent data support the potential efficacy of amivantamab in patients with uncommon EGFR mutations. In a small real-world study, amivantamab monotherapy achieved an ORR of 85.7% (6 of 7 patients) and a disease control rate (DCR) of 100% in this patient subset (26). Similarly, results from the CHRYSALIS-2 study demonstrated comparable efficacy. In that study, treatment-naïve patients and those previously treated with first- or second-generation EGFR-TKIs received combination therapy with amivantamab plus lazertinib (27). In cohort C, which included 105 participants harboring uncommon mutations—G719X (56%), L861X (26%), and S768I (23%)—the ORR was 52%, the median duration of response was 14.1 months, and the median PFS was 11.1 months (3). These findings indicate clinically meaningful antitumor activity of the amivantamab–lazertinib combination. However, direct evidence demonstrating the efficacy of amivantamab plus chemotherapy in patients with uncommon EGFR mutations is currently lacking.

In the present case, amivantamab plus lazertinib could have been an alternative therapeutic option at the time of the latest progression. However, this combination carries a potential risk of deteriorating the patient’s performance status due to previously reported EGFR-related adverse events, such as diarrhea and skin rash. Furthermore, the financial burden was a critical consideration, as neither drug was reimbursed in our country at that time. Although disease progression had occurred after prior pemetrexed/carboplatin therapy, the previous response had been notably durable. We hypothesized that the intervening ABCP treatment might have altered the clonal composition of the tumor, potentially re-sensitizing it to pemetrexed and carboplatin. Considering this clonal evolution and the need to mitigate ‘financial toxicity,’ we opted for amivantamab combined with a rechallenge of pemetrexed/carboplatin. The profound response to this regimen suggests that amivantamab may synergistically overcome established chemotherapy resistance by simultaneously targeting the EGFR/MET pathways and enhancing immune-mediated tumor clearance. While further investigations are warranted, our case highlights that amivantamab plus chemotherapy represents a feasible and highly effective salvage strategy for this high-risk population.

It is important to note that the MET expression or amplification status was not formally assessed in the current case, as tissue availability was limited for additional molecular studies at the time of the third progression. However, data from the MARIPOSA-2 trial demonstrated that the efficacy of the amivantamab combination was superior to the control group regardless of MET status. This suggests that amivantamab effectively inhibits not only specific resistance mechanisms such as MET amplification but also a broad range of bypass signaling pathways mediated through the EGFR axis. Despite the lack of information regarding $MET$ status at the initiation of the combination, our case supports the clinical feasibility of this regimen in real-world practice, even in the absence of exhaustive molecular profiling.

Interestingly, in our case, the ABCP regimen (atezolizumab, bevacizumab, paclitaxel, and carboplatin) combination regimen also produced a durable clinical response. In a subgroup analysis of the phase 3 IMpower150 trial, this quadruple combination demonstrated clinically meaningful survival benefits in patients with sensitizing EGFR mutations (10). In that study, the ABCP regimen yielded a superior median PFS of 10.2 months compared with 6.9 months in the BCP arm. Similarly, the median OS was significantly longer in the ABCP group than in the BCP group (29.4 months vs. 19.1 months; HR 0.61). Notably, this quadruplet regimen provided a significant survival advantage in patients with baseline hepatic metastases compared with the BCP arm in both PFS (8.2 months vs. 5.4 months) and OS (13.3 months vs. 9.4 months) (10). More recently, the phase 3 ATTLAS study, conducted in Korean patients with EGFR-mutant NSCLC, demonstrated that ABCP was superior to paclitaxel plus carboplatin in terms of PFS (8.5 months vs. 5.6 months; HR 0.62; p = 0.004) and ORR (69.5% vs. 41.9%) (28). Collectively, these data consistently suggest that the ABCP quadruple regimen may represent a preferred treatment strategy for patients with EGFR-mutant NSCLC who develop hepatic metastases following TKI failure.

EGFR-mutant NSCLC is typically considered an immunologically “cold tumor,” meaning it is less responsive to immunotherapy. However, anti–vascular endothelial growth factor (anti-VEGF) treatment can reverse the immunosuppressive microenvironment induced by prior TKI therapy by promoting dendritic cell maturation and facilitating T-cell infiltration (29). Moreover, as VEGF expression is increased in hepatic metastases compared with other metastatic sites—and because VEGF and EGFR signaling share downstream pathways—dual EGFR–VEGF pathway inhibition is considered one of the most promising strategies to overcome resistance to targeted therapy (30). The clinical response to the ABCP regimen in our case aligns with the aforementioned positive findings from previous studies. Given the biological interplay between VEGF and EGFR, an anti-VEGF–containing combination regimen may represent a feasible treatment option for patients with EGFR-mutant NSCLC, even for those with hepatic metastases.

In conclusion, the present case underscores the potential of amivantamab in combination with chemotherapy as an effective salvage strategy for patients with uncommon EGFR-mutant NSCLC and systemic dissemination. Amivantamab’s dual inhibition of the EGFR and MET pathways, together with its immune-modulating activity, appears to effectively overcome established resistance mechanisms and induce a rapid, durable response even in the presence of extensive hepatic and peritoneal metastases. This report provides a clinical rationale for considering amivantamab-based therapeutic intensification as a feasible option within the evolving landscape of precision oncology for NSCLC. Furthermore, clinicians should endeavor to bridge the gap between emerging evidence and real-world practice by engaging patients and caregivers in shared decision-making, even in unfavorable clinical contexts. A proactive, evidence-driven approach to subsequent treatment—regardless of disease severity—is essential to optimize management and improve patient survival.

## Data Availability

The original contributions presented in the study are included in the article/supplementary material. Further inquiries can be directed to the corresponding author.
